# A Case Report of Anti-TIF1-*γ*Antibody-Positive Dermatomyositis Concomitant with Small Cell Neuroendocrine Carcinoma of the Urinary Bladder

**DOI:** 10.1155/2023/8837463

**Published:** 2023-12-12

**Authors:** Hiroyuki Hounoki, Takafumi Onose, Miho Yamazaki, Ryoko Asano, Satoshi Yamaguchi, Koichiro Shinoda, Kazuyuki Tobe, Akira Noguchi, Kenichi Hirabayashi

**Affiliations:** ^1^First Department of Internal Medicine, University of Toyama, Toyama, Japan; ^2^Department of Diagnostic Pathology, University of Toyama, Toyama, Japan

## Abstract

Small cell neuroendocrine carcinoma is rare among urinary bladder cancer types, and to date, there are no case reports of concurrent antitranscriptional intermediary factor 1-*γ*antibody-positive dermatomyositis. We describe the case of a 69-year-old Japanese man who presented with elevated creatine kinase levels and haematuria on medical examination. Approximately one month later, he developed dysphagia. Laryngoscopy confirmed laryngeal dysfunction. He also presented with muscle weakness and a skin rash. Magnetic resonance imaging of the upper extremities suggested bilateral brachial muscle myositis. He was diagnosed as having dermatomyositis and was later found to be positive for antitranscriptional intermediary factor 1-*γ* antibody. Computed tomography revealed an intravesical space-occupying lesion and right iliac lymphadenopathy, suggesting urinary bladder cancer. The patient was admitted to our hospital for treatment. Urinary bladder biopsy confirmed small cell neuroendocrine carcinoma because tumour cells were positive for synaptophysin, CD56, and chromogranin A. Thus, the patient was diagnosed as having an antitranscriptional intermediary factor 1-*γ*antibody-positive dermatomyositis concomitant with urinary bladder small cell neuroendocrine carcinoma. The patient was treated with glucocorticoid and intravenous immune globulin therapy for dermatomyositis. Radiotherapy was selected for the carcinoma. Although muscle weakness and skin symptoms improved with treatment, dysphagia persisted. Furthermore, expression of the transcriptional intermediary factor 1-*γ* protein in tumour cells was also confirmed by immunohistochemistry, but the significance is unknown. It should be noted that antitranscriptional intermediary factor 1-*γ*antibody-positive dermatomyositis can occur concomitantly with such a rare malignancy.

## 1. Introduction

Polymyositis/dermatomyositis is an inflammatory muscle disease that primarily causes inflammation of the muscles at the proximal extremities, trunk, and neck through an autoimmune mechanism, which results in muscle pain and weakness. Polymyositis is clinically characterised by muscle symptoms without skin symptoms, whereas dermatomyositis is characterised by typical skin symptoms, such as Gottron's sign and heliotrope eruption [1]. Recently, multiple myositis-specific autoantibodies associated with polymyositis/dermatomyositis have been identified. These antibodies can serve as markers to predict the clinical features and disease course [2–5].

Malignancy complications are among the clinical features of polymyositis and dermatomyositis, with a reported rate of 10%–30%, although different rates have also been observed [6]. Importantly, antitranscriptional intermediary factor 1-*γ* (TIF1-*γ*) antibodies are known to be strongly associated with malignant tumours [7–10]. Thus, adult cases positive for anti-TIF1-*γ* antibodies may be concomitant with malignant tumours at a high rate. In order to reach a deeper understanding of this association, a close examination of malignant tumours is necessary. Some of their most common sites of occurrence are the breast, large intestine, ovaries, lungs, and nasopharynx [11–13].

In addition, there are few reports of dermatomyositis concomitant with urinary bladder cancer (UBC) [14–17]. UBC can be classified into different subtypes, among which small cell neuroendocrine carcinoma (SCNEC) is rare. Within the scope of our search, anti-TIF1-*γ*antibody-positive dermatomyositis concomitant with SCNEC of UBC has never been reported. Hence, we report a very valuable case in which we confirmed the expression of TIF1-*γ* protein in tumour cells.

## 2. Case Presentation

**Figure 1:**
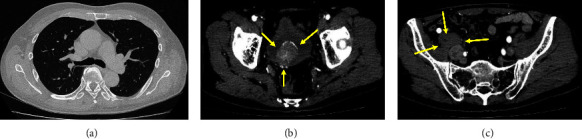
Imaging findings on admission. (a) A chest computed tomography (CT) scan showed no interstitial pneumonia. (b) CT from the abdomen to the pelvis showed a mass lesion protruding from the dorsal wall of the urinary bladder into the lumen, with calcification at the margin (arrows). (c) Swelling thought to be metastasis was observed in the right iliac lymph node (arrows).

A 69-year-old Japanese man was found to have elevated creatine kinase (CK) and haematuria during a medical examination. Approximately one month later, he rapidly developed dysphagia and was admitted to a general hospital. Laryngoscopy revealed no organic lesions. However, dysphagia due to poor laryngeal elevation was observed. Gastrointestinal endoscopy revealed no organic lesions other than atrophic gastritis. Head computed tomography (CT) findings showed no organic abnormalities; however, muscle weakness was noted in both upper extremities, together with Gottron's sign in both fingers. Magnetic resonance imaging (MRI) revealed a diffuse increase in signal intensity in both upper arm muscles, suggesting myositis. A skin biopsy of the upper extremities revealed empty cell degeneration and lymphocytic infiltration at the base of the epidermis, lymphocytic infiltration around the blood vessels in the upper dermis, degeneration of collagen fibres, and mucin deposition in the dermis, all consistent with dermatomyositis. CT findings showed no interstitial pneumonia; however, a space-occupying lesion in the urinary bladder and right iliac lymphadenopathy was observed, which suggested UBC and lymph node metastasis (Figure 1). The patient was clinically diagnosed as having dermatomyositis concomitant with UBC, and glucocorticoid therapy was initiated.

The patient was found to be positive for anti-TIF1-*γ* antibody. He was transferred to our hospital for further evaluation and treatment. Physical examination revealed normal body temperature and oxygen saturation of 97% in room air. No rales were heard in the chest. Erythema was observed on the face, scalp, and neck, whereas epidermal erosion and ulceration were observed on the left axilla. Gottron's sign was observed on the fingers, erythematous keratoses were scattered around the ankle joints, and scratch dermatitis was observed on the back. Weakness of extremity muscles was also observed. There was no lateral difference in the deep tendon reflexes. Laboratory findings on admission (Table 1) revealed elevated CK, neutrophil count, C-reactive protein level, and hypoalbuminaemia. The anti-TIF1-*γ* antibody titre was elevated, whereas KL-6 was not. Urinalysis revealed occult blood and atypical cells.

**Figure 2:**
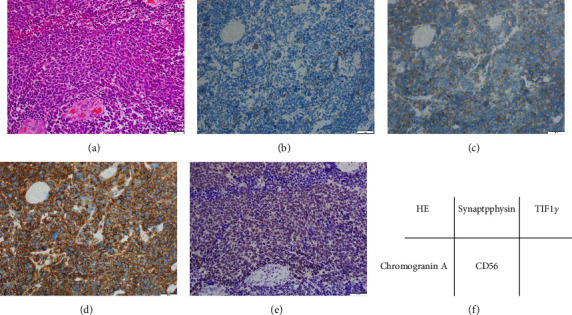
Images of small cell neuroendocrine carcinoma (SCNEC) of the urinary bladder. (a) SCNEC showing high-grade nuclei without nucleoli and with scant cytoplasm by haematoxylin-eosin staining. (b) Tumour cells are partially positive for chromogranin A. (c) Tumour cells are weakly positive for synaptophysin. (d) Tumour cells are diffusely positive for CD56. (e) Tumour cells are strongly positive for TIF1-*γ* while surrounding normal cells are negative. (f) Corresponding table of tissue staining.

In the urinary bladder, a mass lesion was observed with a calcified margin, and the right iliac lymph node was swollen. CT findings suggested UBC with lymph node metastasis (Figure 1). A biopsy of the urinary bladder was performed, and the findings indicated SCNEC because tumour cells were positive for synaptophysin, CD56, and chromogranin A. The results of haematoxylin-eosin staining and immunohistochemical analysis of tumour cells are shown in Figure 2. The patient was finally diagnosed with anti-TIF1-*γ*antibody-positive dermatomyositis concomitant with SCNEC of the urinary bladder. The dermatomyositis was treated with glucocorticoid and intravenous immune globulin therapy, and palliative radiation therapy was administered for UBC. Radiation therapy with a total dose of 60 Gy was administered to the target primary invasive UBC and pelvic lymph node metastases. Considering the patient's general condition, chemotherapy was not administered. After treatment, CK normalised, and muscle weakness and eruption improved, but dysphagia persisted. The value of anti-TIF1-*γ* antibodies was 137 before the administration of glucocorticoid or immunoglobulin and was 93 about 3 months after treatment.

## 3. Discussion

Polymyositis/dermatomyositis is an autoimmune inflammatory myopathy. One of its well-known clinical characteristics is the frequent cooccurrence with malignant tumours. Although there are differences in the literature, the rate of this cooccurrence is 10–30% [6]. In line with this, polymyositis/dermatomyositis is considered a heterogeneous condition with various pathological scenarios. Many myositis-specific antibodies have been identified to date [2–5]. Classification of myositis based on myositis-specific antibodies is considered physiologically relevant because each of the different antibody profiles is associated with a certain subset of polymyositis/dermatomyositis clinical features. Among myositis-specific antibodies, the anti-TIF1-*γ* antibody was originally named anti-p155/140 antibody because TIF1-*γ* is a 155 kDa protein that precipitates in patient serum. In contrast, a study by Fujimoto et al. showed that the 140 kDa protein was actually TIF1-*α* [18], another one of the four TIF1 family proteins. TIF1-*γ* was reported to be a tumour suppressor gene [19], with structural similarity with tripartite motif-containing 33 (TRIM33) [20, 21]. Anti-TIF1-*γ* antibodies are positive in approximately 20% to 35% of juvenile dermatomyositis and are rarely detected in polymyositis [22, 23]. Approximately 7–31% of adult dermatomyositis cases have been reported to be positive. This dermatomyositis [19], as anti-TIF1-*γ* antibodies in adult dermatomyositis, is known to have a strong correlation with malignancies. Indeed, a meta-analysis published in 2012 revealed that anti-TIF1-*γ* antibody had a sensitivity of 78% (95% CI: 45–94%) and specificity of 89% (95% CI: 82–93%) for dermatomyositis complicated by malignant tumours [24]. A meta-analysis published in 2014 reported a relative risk of 5.57 in tumour prevalence (95% CI: 2.91–10.65) [25]. The particularities of the present case, which also had UBC, lead to a novel association concerning this cancer type. Breast cancer was the most common cancer type and most frequent malignant tumour associated with anti-TIF1-*γ*antibody-positive dermatomyositis (33%), followed by ovarian cancer (19%) and lymphoma (14%) [25]. To the best of our knowledge, a total of 26 reports of UBC associated with dermatomyositis have been published so far, of which only one was categorised as SCNEC of UBC [15]. Furthermore, there have been no previous reports evaluating anti-TIF1-*γ* antibodies in any of these reports. According to Pang et al. the most common histologic types of UBC were urothelial carcinoma (91.4%), squamous carcinoma (1.9%), and adenocarcinoma (1.8%) [26]. SCNEC of the urinary bladder is an extremely rare but aggressive entity constituting less than 0.7% of all urinary bladder tumours [27, 28]. In the literature, the sensitivity of progastrin-releasing peptide (ProGRP) in SCNEC is 76% in the lungs and 54% in nonlung areas, and the sensitivity of neuron-specific enolase (NSE) in SCNEC is 62% [29]. In some cases, both ProGRP and NSE do not increase, as observed in this case. Juvenile dermatomyositis, contrary to adult, is not associated with malignant tumours [19]. A frequent finding in anti-TIF1-*γ*antibody-positive dermatomyositis is an eruption, which is often extensive and prominent in children and adults and may present with blistering and erythroderma. Eruptions tend to be distributed in exposed areas. Conversely, less common findings are interstitial lung disease, Raynaud's phenomenon, and arthritis [30]. In our case, the eruption was prominent; however, interstitial pneumonia, Raynaud's phenomenon, and arthritis were not observed, which is consistent with a previous report [30]. Mugii et al. have reported an association between anti-TIF1-*γ*antibody-positive dermatomyositis and dysphagia. In this study, 13 patients with dysphagia, who showed positivity for specific autoantibodies detected in dermatomyositis, anti-U1-RNP antibody in mixed connective tissue disease, and anti-SS-A antibody in Sjögren's syndrome, were analysed, together with 79 patients without dysphagia. A significant difference was observed for dysphagia only in the anti-TIF1-*γ*antibody-positive dermatomyositis group [31]. In our case, dysphagia developed rapidly, approximately one month after receiving the medical examination, which supports the report by Mugii et al. Furthermore, in the present case, laryngeal endoscopy confirmed poor elevation of the larynx, which was thought to be due to inflammation of the muscles involved in swallowing. A candidate explanation for this high cooccurrence of TIF1-*γ* antibodies in dermatomyositis and malignancies is that abnormal TIF1-*γ* is observed in cancer cells and induces the production of anti-TIF1-*γ* antibodies, which in turn contribute to their own destruction. However, anti-TIF1-*γ* antibodies can also have cross-reactivity with the nonmutated TIF1-*γ* antigen expressed in keratinocytes and myoblasts, resulting in dermatomyositis [32]. Considering this, the expression frequency of TIF1-*γ* in the muscle groups related to swallowing and dysphagia is presumably related. However, to our knowledge, no literature reporting the expression frequency of TIF1-*γ* in muscle groups related to swallowing was found. The fact that TIF1-*γ* staining was observed in the nuclei of tumour cells is consistent with the report by Motegi et al. [33]. A limitation of these findings is that we could not determine whether the TIF1-*γ* observed in the tumour cells was the abnormal TIF1-*γ* antigen commonly expressed in cancer cells, or the original one.

Herein, we report a case of anti-TIF1-*γ*antibody-positive dermatomyositis concomitant with SCNEC in the urinary bladder. Urinary bladder SCNEC is a rare condition. No reports exist of an immunohistological evaluation of SCNEC of the urinary bladder in a case with a cooccurring anti-TIF1-*γ*antibody-positive dermatomyositis, which highlights the novelty and physiological relevance of this case. Though the significance of high expression of the TIF1-*γ* protein in tumour cells is unknown in this case, it should be noted that anti-TIF1-*γ*antibody-positive dermatomyositis can occur concomitantly with such a rare malignancy.

## Figures and Tables

**Table 1:** Laboratory findings on admission.

*Urinalysis*	
Urine protein	(2+)
Occult blood	(3+)
Urine WBC	Undecidable
Urine RBC	>100 HF
Atypical cells	(+)

*Complete blood count*	
WBC	10690 *μ*L
Neutrophil	9170 *μ*L
RBC	4.17 × 106 *μ*L
Hemoglobin	12.4 g/dL
Hematocrit	37.0%
Platelet	25.0 × 104 *μ*L

*Immunology*	
FANA (homo 1; 40, speckled 1; 80)	1; 80
Anti-dsDNA Ab (RIA)	≦1.7 U/mL
Anti-RNP Ab	<2.0 U/mL
Anti-Sm Ab	<0.8 U/mL
Anti-SS-A Ab	<0.4 U/mL
Anti-SS-B Ab	<0.4
Anti-ARS Ab	<5.0
Anti-Jo-1 Ab	<0.3
Anti-MDA 5 Ab	<4.0
Anti-Mi-2 Ab	<5.0
Anti-TIF1-*γ* Ab (normal range <32)	137

*Biochemistry*	
TP	6.1 g/dL
Alb	3.3 g/dL
BUN	26.7 mg/dL
Creatine	0.86 mg/dL
AST	88 U/L
ALT	76 U/L
LDH	475 U/L
CK	501 U/L
TSH	1.44 *μ*U/L
FT3	1.4 pg/mL
FT4	1.6 ng/mL
KL-6	349.8 U/mL

*Tumour marker*	
ProGRP (normal range <81)	37.5 pg/mL
NSE (normal range <16.3)	15.3 ng/mL

*Fecal occult blood test*	
Negative study for two consecutive times	

## Data Availability

The data used to support the findings of this study are available from the corresponding author upon request.
